# Cutin Synthesis in Developing, Field-Grown Apple Fruit Examined by External Feeding of Labelled Precursors

**DOI:** 10.3390/plants10030497

**Published:** 2021-03-05

**Authors:** Yiru Si, Bishnu P. Khanal, Leopold Sauheitl, Moritz Knoche

**Affiliations:** 1 Institute of Horticultural Production Systems, Fruit Science Section, Leibniz University Hannover, Herrenhäuser Straße 2, 30419 Hannover, Germany; yiru.si@obst.uni-hannover.de (Y.S.); khanal@obst.uni-hannover.de (B.P.K.); 2 Institute of Soil Science, Leibniz University Hannover, Herrenhäuser Straße 2, 30419 Hannover, Germany; sauheitl@ifbk.uni-hannover.de

**Keywords:** cutin, wax, oleic acid, palmitic acid, *Malus* × *domestica*, cuticle, epidermis, hypodermis

## Abstract

An intact skin is essential in high-quality apples. Ongoing deposition of cuticular material during fruit development may decrease microcracking. Our objective was to establish a system for quantifying cutin and wax deposition in developing apple fruit. Oleic acid (^13^C and ^14^C labelled) and palmitic acid (^14^C labelled) were fed to developing apples and the amounts incorporated in the cutin and wax fractions were quantified. The incorporation of ^14^C oleic acid (C18) was significantly higher than that of ^14^C palmitic acid (C16) and the incorporation in the cutin fraction exceeded that in the wax fraction. The amount of precursor incorporated in the cutin increased asymptotically with time, but the amount in the wax fraction remained about constant. Increasing the concentration of the precursor applied generally increased incorporation. Incorporation in the cutin fraction was high during early development (43 days after full bloom) and decreased towards maturity. Incorporation was higher from a dilute donor solution (infinite dose feeding) than from a donor solution subjected to drying (finite dose feeding) or from perfusion of the precursor by injection. Feeding the skin of a developing apple with oleic acid resulted in significant incorporation in the cutin fraction under both laboratory and field conditions.

## 1. Introduction

Maintaining an intact fruit skin is essential in the production of high-quality fruit of all species. Skin defects impair the cuticle’s barrier properties [1]. They allow uncontrolled passage of substances including water into and out of the fruit, leading to excessive water loss (in the dry) and shrivel or to water uptake (in the wet), cracking and russeting [2,3,4,5,6,7]. In addition, the cuticle’s function as a barrier against invasion by pathogens is also impaired [8,9]. Thus, skin defects compromise both the pre- and postharvest performance of many fruit crop species. A fruit with multiple or severe skin defects is rejected by consumers, so excluded from the marketing chain and so of value only for processing. Hence, skin defects cause serious economic loss to growers.

Microscopic cracks (‘microcracks’) in the cuticle are the first visible sign of many fruit skin defects including the cracking of grapes or sweet cherries [10,11], skin spot in apple [12,13], russeting in apple [2,14,15] or neck shrivel in plum [16].

Microcracking of the cuticle results from excessive skin strain caused by a mismatch between skin surface area growth and cuticular deposition [17,18,19]. During fruit growth, the epidermal and hypodermal cell layers accommodate the increases in skin surface area by cell division (more cells), by cell expansion (larger cells) and by changes in the anticlinal aspect ratio (cell deformation)—they change from portrait to landscape [20]. In contrast, the polymeric cuticle is dragged along and strained by the growth of the underlying cell walls [1]. A concurrent deposition of cutin and an incorporation of wax in the strained cuticle during growth ‘fixes’ the elastic portion of this growth strain [21]. This process leads to a gradient in elastic strain between the outer and inner layers of the cuticle [22] and to a reduced incidence of microcracking. From a fruit-quality perspective, both effects are beneficial, since the likelihood of failure is decreased. Despite their economic importance, not much is known about the factors affecting cuticular deposition in developing fruit, in the field.

Several decades ago, the synthesis and deposition of cuticle was studied in laboratory assays, where radiolabelled fatty acids (^14^C or ^3^H) were applied as precursors to growing leaves and fruit [23,24,25]. Kolattukudy [26] was the first to publish a study on the incorporation of Na-salts of ^14^C-acetate, ^14^C-pentanoate or ^14^C-hexanoate in the wax of leaves of *Brassica oleracea*. These compounds were fed to excised leaves by immersing their petioles in a labelled donor solution. Following an incubation period, the leaf wax was extracted, the extract separated by thin layer chromatography and the amounts of radioactivity associated with the various wax fractions quantified by liquid scintillation spectrometry (LSC) [26]. In the 1970s, ^14^C labelled longer-chain fatty acid precursors, mostly palmitic and oleic acid, were fed to leaves of *Vicia faba* [27,28,29] and to the fruits of apple and grape [30]. Discs were excised from leaves or fruits and were incubated in donor solutions containing the ^14^C labelled precursor. Because the volume of the donor solution was large compared to the volume of the discs, the concentration of the precursor remained essentially constant during an experiment. We therefore refer to this technique as ‘infinite dose feeding’. At the end of the incubation period, the discs were extracted and the wax was removed using a chloroform/methanol solution. The remaining cutin was depolymerized, the constituents obtained separated by thin-layer chromatography. The amount of ^14^C precursor incorporated was determined by LSC. A slightly different approach was taken by Lendzian and Schönherr [25]. In their study ^3^H-labelled palmitic acid was applied as discrete droplets to leaves of *Clivia miniata* Reg. [25]. This system is similar to a finite dose system, where the precursor concentration in the donor droplet changes as penetration of the precursor into the leaves proceeds [31,32]. The incorporation of ^3^H-labelled palmitic acid was quantified after depolymerization of the cutin with BF_3_-methanol. To our knowledge, no studies have been published on cuticle formation under field conditions. Here, the use of ^14^C-labelled precursors is prohibited, so other methods such as ^13^C-labelled precursors must be used instead.

The objective of this study was to develop a system that permits quantification of cuticle, that is, cutin and wax, deposition in intact, growing fruit. We choose developing apple fruit as a model, since (1) apple is an economically very important fruit crop, (2) the apple cuticle is well characterized [21,22,33] and (3) apples continue to deposit cutin and wax throughout their development [17].

## 2. Results

**Figure 1:**
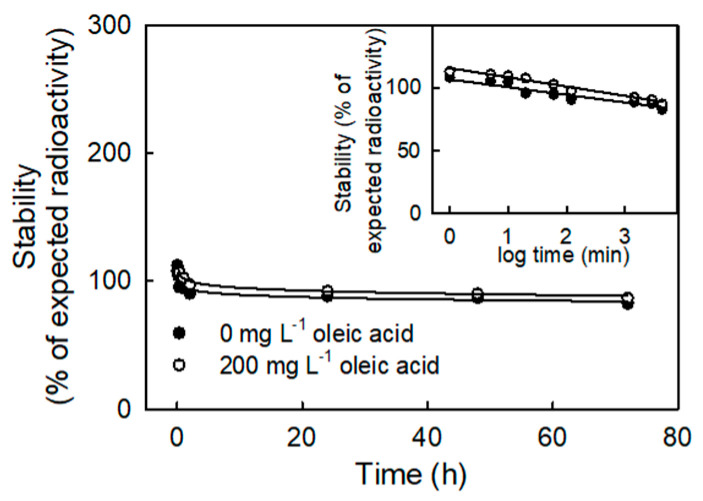
Stability of fatty acid solutions. Time course of change in radioactivity in the lower layers of an oleic acid solution in a scintillation vial without shaking (solution height 12 mm, solution volume 5 mL). Both solutions had 19,269 (±160) dpm total radioactivity and 0 or 200 mg L^−1^ of un-labelled (cold) oleic acid. Time on the *x*-axis is log-transformed and re-plotted in the inset. The values are means ± SEs of five replications.

The supernatant of donor solutions containing ^14^C oleic acid decreased in concentration with time (Figure 1). The decrease followed an exponential pattern. For a typical run, the duration between the final vortexing of the donor solution and the end of the application averaged about 10 min. During this time, the relative decrease in radioactive concentration in the supernatant of the donor solution was about 3%.

**Figure 2:**
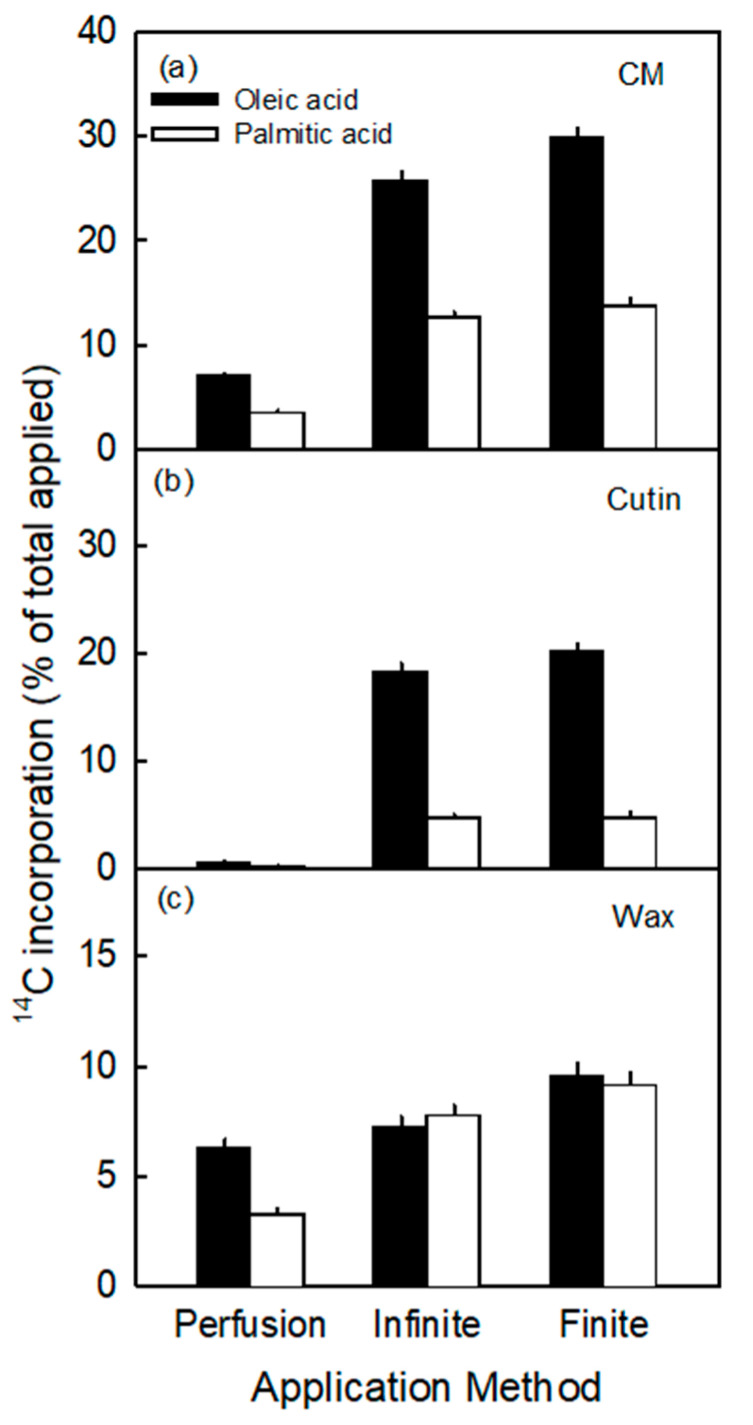
Incorporation of ^14^C (% of total applied) of oleic acid and palmitic acid in cuticular membrane (CM) (**a**), cutin (**b**) and wax (**c**) as affected by application method. ^14^C labelled oleic or palmitic acid was applied to the excised exocarp segments (ES) of young fruit of ‘Idared’ apples sampled at 43 days after full bloom (DAFB). The values are means ± SEs of 15 replications.

Incorporation in the cuticular membrane (CM) and the cutin fractions was significantly higher following application of ^14^C oleic acid (C18) as compared to ^14^C palmitic acid (C16) (Figure 2). Infinite and finite dose feeding resulted in higher incorporations than perfusion. In contrast to the cutin fraction, there was little difference between the two precursors for the wax fraction. Based on the higher incorporation, oleic acid was selected as the precursor for all subsequent experiments.

**Figure 3:**
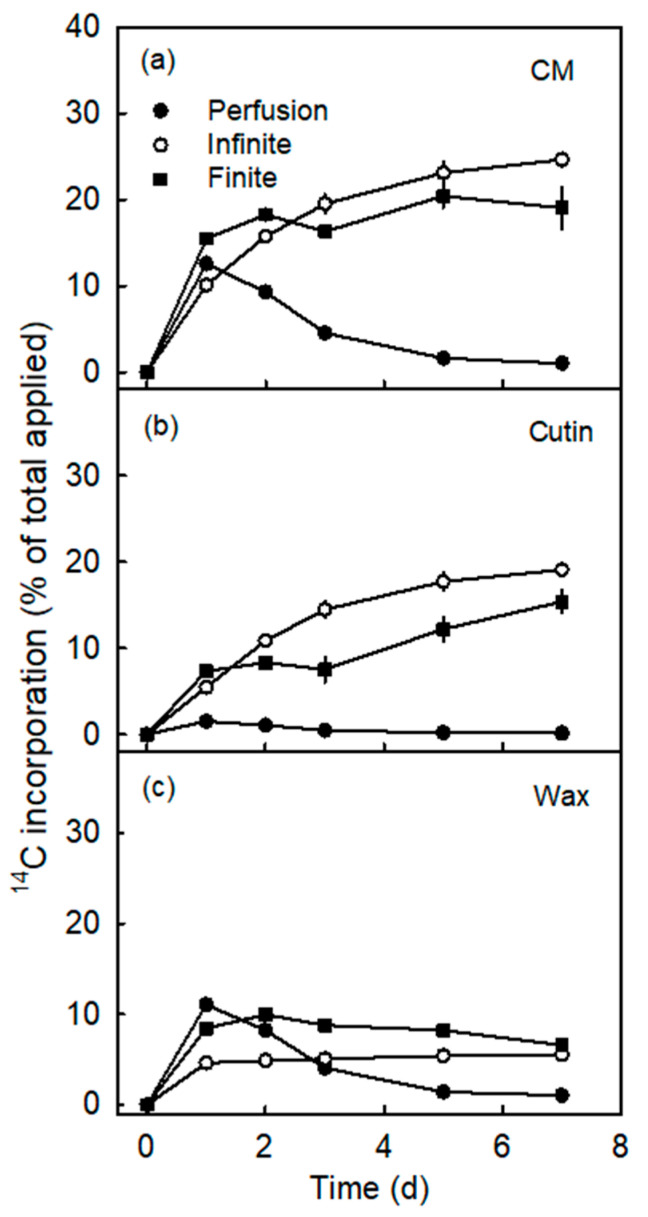
Time course of ^14^C incorporation (% of total applied) in cuticular membrane (CM) (**a**), cutin (**b**) and wax (**c**). ^14^C labelled oleic acid was applied to the excised exocarp segments (ES) of young ‘Idared’ apples, sampled at 62 days after full bloom (DAFB), using three different application methods. The values are means ± SEs of 15 replications.

The amount of ^14^C oleic acid incorporated in the CM and the cutin fraction following infinite dose feeding increased asymptotically with time. The radioactivity associated with the wax fraction was nearly constant and essentially independent of time (Figure 3). Incorporation following finite dose feeding was rapid into the CM and the cutin fractions during the drying of the donor solution (within 1 d). Thereafter, incorporation slowed down and proceeded at a low rate. Incorporation in wax decreased slowly after 2 d of finite dose feeding. There was higher incorporation in wax after finite dose feeding than after infinite dose feeding. Following perfusion, incorporation in the CM and the wax fractions decreased with time. The incorporation in the cutin fraction was only marginal. After 7 d, incorporation into the CM and the cutin fraction was highest in infinite dose feeding, followed by finite dose feeding. The lowest incorporation was measured following perfusion.

**Figure 4:**
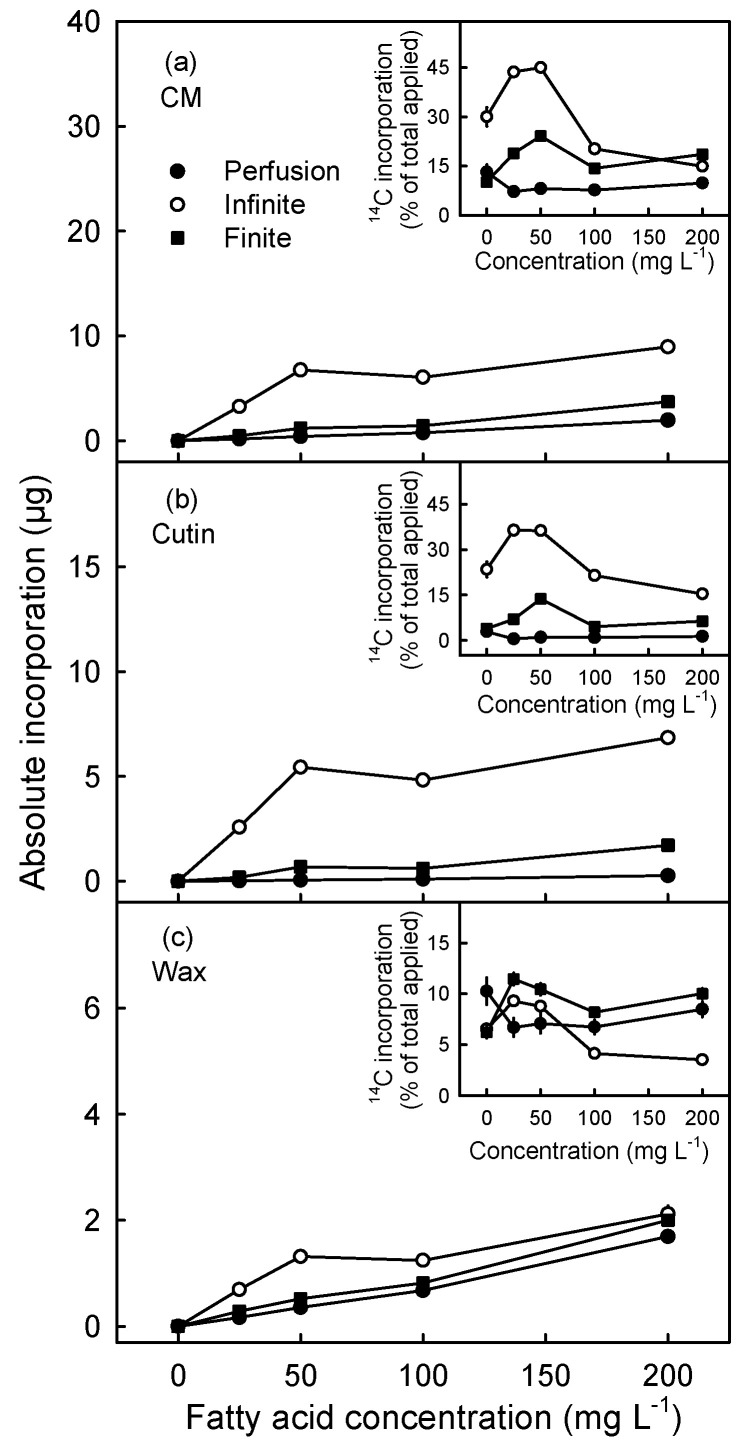
Effect of fatty acid concentration of feeding solution in the incorporation of ^14^C in absolute amount (ng; main figures) and in relative amount (% of total applied; inset figures) in cuticular membrane (CM) (**a**), cutin (**b**) and wax (**c**). ^14^C labelled oleic acid was applied to the excised exocarp segments (ES) of young ‘Idared’ apples, sampled at 83 days after full bloom (DAFB), using three different application methods. The concentrations of fatty acid in the feeding solutions were adjusted using un-labelled (cold) oleic acid. The values are means ± SEs of 15 replications.

Increasing the concentration of oleic acid in the feeding solution increased the amount incorporated in the cuticle, the cutin and the wax fractions (Figure 4). Incorporation was consistently higher after infinite dose feeding, followed by finite dose feeding and by perfusion. On the basis of the percent of mass applied, incorporation into the cuticle and the cutin fractions, but not into the wax fraction, was generally higher following infinite dose feeding.

**Figure 5:**
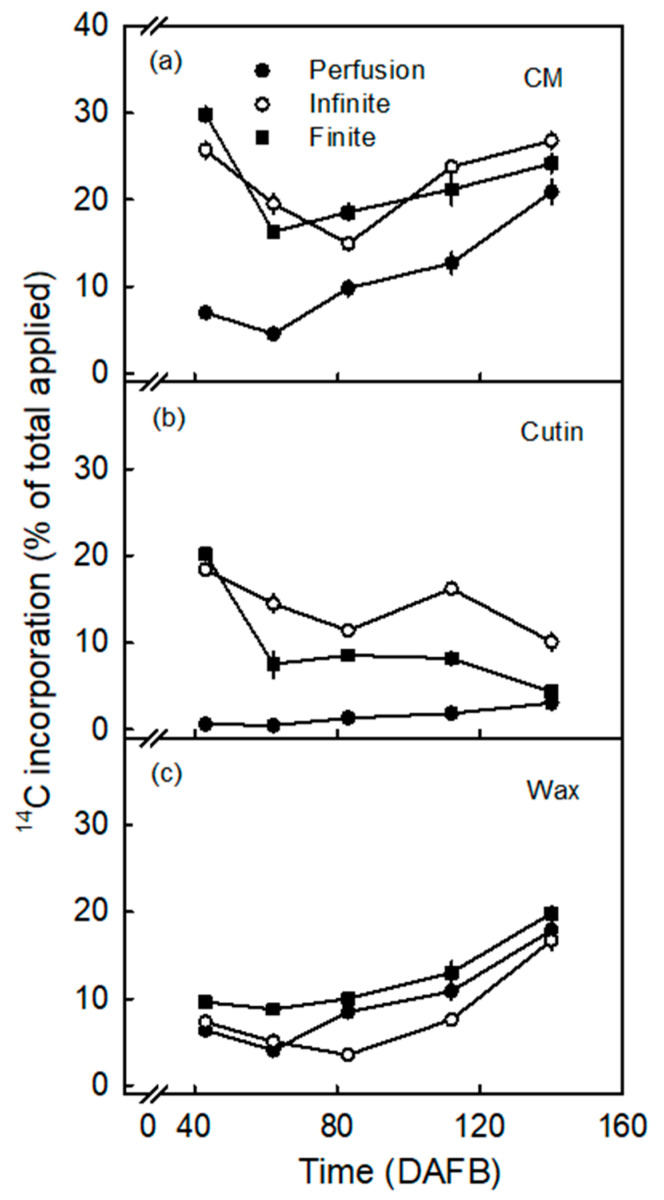
Effect of fruit development stage on ^14^C incorporation (% of total applied) in cuticular membrane (CM) (**a**), cutin (**b**) and wax (**c**). ^14^C labelled oleic acid was applied to the excised exocarp segments (ES) of ‘Idared’ apples, sampled at various stages of development between 43 days after full bloom (DAFB) and maturity (140 DAFB), using three different application methods. The values are means ± SEs of 15 replications.

The developmental time course revealed high incorporation in the cuticle and the cutin fractions during early development and following infinite and finite dose feeding (Figure 5). Incorporation in both fractions then decreased between 43 and 83 days after full bloom (DAFB). For the CM, incorporation increased again towards maturity. There was no consistent change in the cutin fraction after 83 DAFB. This increase in the CM fraction was primarily due to a consistent increase of incorporation in the wax fraction. There was essentially no incorporation following perfusion feeding in the cutin, but incorporation in the CM and the wax fraction significantly increased towards maturity. Incorporation was lowest following perfusion feeding.

The results obtained when feeding developing attached fruit in the field using ^13^C oleic acid and detached fruit from the same trees in the lab using ^14^C oleic acid were generally similar. Incorporation was highest in the CM and the cutin fractions and following infinite dose and finite dose feeding (Table 1). The lowest incorporation was observed using perfusion feeding.

## 3. Discussion

The major findings are: (1) Patterns of incorporation of precursors by the CM reflected patterns of synthesis, deposition and incorporation by the cutin fraction. For the wax fraction we observed what was probably a simple partitioning phenomenon; (2) Incorporation from infinite dose feeding was slightly greater than that from the finite dose feeding. Incorporation from perfusion was consistently the least; (3) Incorporation of oleic acid was greater than that of palmitic acid.

### 3.1. Incorporation into the Cutin Fraction Reflects Synthesis, but Not a Simple Partitioning

It can be argued that the amount of radioactivity associated with the cuticle, in this and earlier studies, resulted from a simple partitioning of the precursors into the cuticle. Indeed, the cuticle is a lipophilic sorption compartment and both precursors are fatty acids [34]. However, several arguments suggest that simple partitioning did not play a significant role.

(I) The incorporation of the precursors in the cutin fraction was highly selective. It consistently favored oleic acid over palmitic acid, despite their very similar octanol/water partition coefficients (Koct/w) (i.e., for oleic acid logarithm of Koct/w is 7.64 and for palmitic acid it is 7.17) [35]. The value of log Koct/w is closely related to the logarithm of the cuticle/water partition coefficient (log Kcut/w) [36]. The two calculated log Kcut/w values are 7.47 for oleic acid and 7.01 for palmitic acid. The measured selectivity of incorporation in the cutin fraction is consistent with the composition of apple fruit cutin where C18 compounds dominate over C16 ones, with ratios ranging from 2.2:1 to 3.4:1 [37,38,39]. This ratio is close to that obtained in our study (range 3.8 and 4.2 for the infinite and finite dose feeding, respectively; Figure 2).
(II) When the cuticle was extracted using a chloroform/methanol solution, the radiolabel associated with the cuticle remained in the cutin polymer. If the two precursors were only partitioned into the cutin, we would have expected both precursors to be extracted by the chloroform/methanol solution and, hence, to end up in the supernatant as part of the wax fraction. However, this was clearly not the case for the cutin fraction.
(III) Kolattukudy and Walton [29] and Kolattukudy et al. [38] reported that externally applied oleic acid converts into 18-hydroxy-C18 or 10,18-dihydroxy-C18 or 9,10,18-trihydroxy-C18 acid and externally applied palmitic acid into 16-hydroxy-C16 or 10,16-dihydroxy-C16 acid before incorporation into the cutin.

The results obtained for the wax fraction differed from those for the cutin fraction indicating that a clear preference and selectivity for one or other of the two precursors was absent. After feeding the two fatty acids, the ratio of the radioactivity in the wax fraction was close to 1 (range 0.9 and 1.0 for the infinite and finite dose feeding, respectively; Figure 2). This is not surprising given: (1) the similarity in lipophilicity between the two precursors as indexed by their similar log Koct/w and log Kcut/w values, (2) the diversity of pathways for the syntheses of individual constituents that contribute to the so-called wax and (3) the monomeric character of the wax. In contrast to the polymeric cutin, the wax fraction is monomeric. Hence, it is technically impossible to distinguish a radioactive fraction of precursors that resulted from simple partitioning of the fatty acid into a lipoidal wax fraction, from a fraction that resulted from metabolic modifications of the precursors and their incorporation into newly-synthesized constituents of the apple fruit wax. Because of these restrictions, our subsequent discussion is focused on the cutin fraction.

### 3.2. Incorporation Was Higher Following Infinite Dose Feeding Than Finite Dose Feeding—Both Methods Were Superior to Perfusion

Incorporation following infinite dose feeding was slightly higher than that following finite dose feeding. Two factors may be involved. First, in finite dose feeding, a dry deposit forms after evaporation of the water solvent. While the concentration of the precursor in the dry deposit is expected to be high, the mobility of the precursor may be limiting after drying due to the absence of a solvent. This would not be the case in infinite dose feeding, where the concentration of the precursor is markedly lower and probably decreases slightly due to penetration and incorporation. The continuing presence of the solvent ensures the precursor remains available for uptake in infinite dose feeding. Second, in finite dose feeding, during the drying of the donor solution the deposit formed does not form a uniform layer over the interface area originally wetted. Instead, as the donor solution dries, it contracts to form a deposit of non-uniform thickness. Hence, the interface area covered by the dried deposit is markedly smaller than the initial contact area between donor solution and fruit surface at the time of application. These two factors are likely to contribute to the slightly lower uptake and incorporation of precursor from the finite, as compared to the infinite dose system.

Incorporation following perfusion was consistently the least. In perfusion, the donor solution containing the precursor is injected into the fruit. The tissue in contact with the precursor is largely sub-epidermal and sub-hypodermal parenchyma, rather than epidermal, whereas the precursors are metabolized within the epidermal layer [7,40,41]. These cell layers contain plastids [42] which are the sites of fatty acid synthesis and, hence, of the precursors of the cutin monomer [41,43,44,45]. This explanation most likely accounts for the low incorporation following perfusion by injection, as compared to either infinite or finite dose feeding where the precursors penetrate directly into the cell layers responsible for the synthesis of cutin, namely the epidermal and hypodermal layers.

### 3.3. Incorporation of Oleic Acid Exceeds That of Palmitic Acid

The proportion of the C16 and C18 monomers in cutin varies between plant species, between organ types, and with organ age. The 9- or 10,16-dihydroxyhexadecanoic acid and the 16-hydroxyhexadecanoic acid of the C16 family and the 18-hydroxy-9,10-epoxyoctadecanoic acid and the 9,10,18-trihydroxyoctadecanoic acid of the C18 family are the dominating monomers of the cutins of various plant species [23]. It is reported that the C16 family of monomers predominates in the cuticles of young and rapidly-expanding plant organs. The proportion of the C18 monomers increases during organ development. Thus, the cutin of a mature organ with a thicker cuticle, contains a larger portion of the C18 monomers [24,29,46]. Also, in apple fruit cutin, higher proportions of C18 monomers than C16 monomer have been reported [37,39]. Our findings are consistent with these reports. According to Kolattukudy and Walton [29] and Walton and Kolattukudy [47], the conversion of exogenously applied palmitic acid into 10,16-dihydroxy-C16 was lower in old leaves than in young leaves of *Vicia faba*. This observation further helps explain the lower incorporation of palmitic acid as compared to oleic acid in apple fruit cutin, since our experiments were all carried out on older fruit, sampled at or later than 40 DAFB.

### 3.4. Conclusions

The results demonstrate that feeding the skin of developing apple fruit with oleic acid results in the incorporation of this precursor into the cutin fraction. Thus, the procedure described herein allows quantification of the deposition of cutin on developing apple fruit using labelled (radioactive or stable isotope) precursors. The precursor of choice for field experimentation is ^13^C-labelled oleic acid. The amount of ^13^C-incorporation is analyzed by mass spectrometry. Our results offer proof that infinite or finite dose feeding is superior to perfusion, which results in a low rate of incorporation. The procedure described herein can be used in future studies to monitor cuticle deposition in the developing fruit of apple and, most probably, other fruit crops under field conditions.

## 4. Materials and Methods

### 4.1. Plant Materials

Developing ‘Idared’ apples (*Malus* × *domestica* Borkh.) were sampled in an experimental orchard of the Leibniz University at Ruthe, Germany (lat. 52°14′ N, long. 9°49′ E). Trees were grafted on M9 rootstocks and cultivated according to current regulations for integrated fruit production.

For the in vitro laboratory assays, ^14^C-labelled precursor fruit were sampled randomly from 150 trees in two adjacent rows. Fruits were immediately transferred to the laboratory where all experiments involving radiolabelled compounds were conducted. For the in vivo assays involving ^13^C-labelled precursor fruit remained attached to the tree and the whole experiment was conducted in the field.

### 4.2. In Vitro Laboratory Assay Using ^14^C

#### 4.2.1. Preparation of Donor Solutions

Donor solutions were prepared usually containing 200 mg L^−1^ of unlabelled (cold) oleic acid (≥99%; Sigma-Aldrich, Deisenhofen, Germany) or palmitic acid (≥99%; Sigma-Aldrich). A surfactant was added at a final concentration of 0.05% (Glucopon 215 UP/MB; BASF SE, Ludwigshafen, Germany). The solution was vortexed for at least 3 min. Thereafter, donor solutions were spiked using ^14^C labelled oleic acid (specific activity 2.2 GBq mmol^−1^, radiochemical purity > 97%; PerkinElmer, Boston, MA, USA) or palmitic acid (specific activity 2.1 GBq mmol^−1^, radiochemical purity ≥ 98%; Moravek Biochemicals, Brea, CA, USA). Both fatty acids were carboxyl labelled. Subsequently, solutions were again vortexed for 3 min. The stability of the donor solutions was checked by sampling the donor solutions repeatedly over time. Aliquots (10 µL) were taken and 3 mL of scintillation liquid (Ultima Gold XR; PerkinElmer, Boston, MA, USA) was added. Samples were radioassayed by liquid scintillation counting (LS 6500; Beckman Coulter Inc., Brea, CA, USA).

#### 4.2.2. Feeding Epidermal Skin Sample with ^14^C-Oleic and ^14^C-Palmitic Acid

Epidermal skin samples (ES) were excised from the equatorial region of the fruit using a cork borer (17 mm inside diameter). The ES were cut to a uniform thickness of about 2–3 mm.

For both the infinite and finite dose feeding, dosing vials were prepared and mounted on the fruit surface (see Figure S1). Briefly, the dosing vials were cut to a 14 mm length from the central portion of a 10 mL polyethylene centrifuge tube (14 mm diameter; Carl Roth, Karlsruhe, Germany). These sections were mounted on the surface of the ES using a non-phytotoxic silicon rubber (Dowsil™ SE 9186 Clear Sealant; Dow Toray, Tokyo, Japan). The ES with tubes attached were placed individually in glass jars that provided a high humidity environment to prevent desiccation during feeding. For the dosing by perfusion, the ES remained without tube and were placed cuticle down in the glass jars.

The ^14^C labelled donor solution was fed to the ES in three different ways:

(1) In the finite dose method, 100 µL of donor solution containing 3 × 10^6^ dpm mL^−1^ radioactivity was applied to the tube on the ES. After feeding, the glass jar was left open to allow evaporation of the water from the feeding solution. Within 15 h of feeding, the donor solution had formed a macroscopically visible dry deposit on the ES. Thereafter, the glass jar was closed.
(2) In the infinite dose method, 300 µL of donor solution containing 1 × 10^6^ dpm mL^−1^ radioactivity was applied to the tube on the ES. Immediately after feeding the glass jar was closed.
(3) In the perfusion method, 100 µL of donor solution containing 3 × 10^6^ dpm mL^−1^ radioactivity was applied directly to the cut surface of the ES. This application mimicked a perfusion of donor solution by injection as performed in experiments using intact fruit attached to a tree in the field.

For all application methods, a water vapor saturated atmosphere was maintained inside the glass jar by adding a piece of moist tissue paper and by closing the jar using a lid. The feeding experiments were carried out at 22 °C. Unless otherwise indicated, the donor deposit (finite dose) or solution (infinite dose) remained visible on the surface of the ES for 72 h.

#### 4.2.3. Sample Preparation and Analysis of Radioactivity

At the end of the feeding period, the donor solution remaining on the ES surface in the infinite setup was collected in a scintillation vial using a pipette. This fraction is referred to as the ‘donor’ (Fraction ‘Donor’).

Thereafter, the outer surface of the ES of the finite and infinite dose setup was rinsed six times with 1% glucopon surfactant solution (300 µL per rinse). The tube was removed from the ES. Subsequently, the tube was rinsed with surfactant solution. All rinse solutions were combined in a scintillation vial. Any residue that may have remained on the surface following the rinsing procedure and that may have been trapped between crystals of epicuticular wax on the surface of the ES was stripped using cellulose acetate stripping [48]. Viscous solutions of cellulose acetate were prepared in acetone and painted on the outer surface of the ES. After drying for 30 min the cellulose acetate solution had hardened and formed a continuous film. The film was peeled off from the surface and dissolved in 1.5 mL acetone in a scintillation vial. The radioactivity in the cellulose acetate strip plus that in the rinse solutions was combined in a ‘rinse’ fraction (Fraction ‘Rinse’).

To separate the cuticular membrane (CM) from adhering tissue of the ES, the discs were incubated in 50 mM citric acid buffer solution (pH 4.0) containing pectinase (90 mL L^−1^, Panzym Super E flüssig; Novozymes A/S, Krogshoejvej, Bagsvaerd, Denmark), cellulose (5 mL L^−1^, Cellubrix L; Novozymes A/S, Krogshoejvej, Bagsvaerd, Denmark). Sodium azide (NaN_3_) was added at a final concentration of 30 mM to avoid microbial growth. Following isolation, CMs were rinsed with deionized water, dried and dewaxed by incubating in 2.0 mL chloroform/methanol (1:1, *v*/*v*) for 16 h at room temperature. Dewaxed CMs (DCMs/cutin) were rinsed with 0.5 mL of chloroform/methanol solution. The chloroform/methanol extract represents the wax fraction (Fraction ‘Wax’). The DCM was depolymerized in tissue solubilizer (Biolutes S; Zinsser Analytic, Frankfurt, Germany) in a scintillation vial for 16 h (Fraction ‘Cutin’).

The isolation solution containing the digested tissue was filtered through cellulose filter paper (Whatman 595; GE Healthcare, Buckinghamshire, UK) and the filtrate collected in a scintillation vial. The residue plus filter paper were dried and subsequently oxidized (OX300; Zinsser Analytic von Gardner Denver, Eschborn, Germany). The radioactivity present in the filtrate plus that in the residue on the filter paper represents the radioactivity in the tissue fraction (Fraction ‘Tissue’).

The radioactivity in the different fractions (Donor, Rinse, Wax, Tissue, and Cutin) was quantified by liquid scintillation counting. A fraction ‘CM’ was calculated as the sum of the cutin plus the wax fractions. Corrections were made for quenching and oxidizer efficiency where appropriate. Recovery of radioactivity was calculated by dividing the sum of radioactivity in all fractions by the amount of radioactivity applied. For a representative data set the recovery of radioactivity averaged 89.1 ± 1.3%, 85.6 ± 1.1% and 47.7 ± 0.9% for the finite, infinite and perfusion feeding, respectively. The number of replicates for each method was 15.

### 4.3. Experiments

The time course of the uptake and incorporation of ^14^C labelled oleic acid was established using finite, infinite and perfusion feeding. The amount of uptake and incorporation was quantified at 0, 1, 2, 3, 5 and 7 d after application. The experiment was conducted using fruit sampled at 62 days after full bloom (DAFB). The number of replicates was 15.

The concentration response of uptake and incorporation of ^14^C labelled oleic acid was quantified. Dosing solutions were prepared at concentrations of 0, 25, 50, 100 and 200 mg L^−1^. The ES were sampled 3 d after application. The experiment was conducted using fruit sampled at 83 DAFB. The number of replicates was 15.

Uptake and incorporation of ^14^C-labelled oleic acid and ^14^C-labelled palmitic acid was compared in fruit sampled at 43 DAFB. Uptake and incorporation of ^14^C-labelled oleic and palmitic acid were quantified 3 d after application. The number of replicates was 15.

The developmental time course was established at 43, 62, 83, 112 and 140 DAFB. Uptake and incorporation of ^14^C-labelled oleic acid was quantified 3 d after application. The number of replicates was 15.

### 4.4. In Vivo Assay Using ^13^C

#### 4.4.1. ^13^C Fatty Acid Solution Preparation

Uniformly ^13^C-labelled oleic acid (>95% purity) and uniformly labelled palmitic acid (>98% purity) were obtained from Larodan (Larodan AB, Solna, Sweden). Donor solutions were prepared at concentrations of 300 mg L^−1^ in 0.05% surfactant solution (Glucopon 215 UP/MB; BASF SE, Ludwigshafen, Germany). Immediately after preparation, the solutions were vortexed for a minimum of 3 min and again shortly before the application in the field.

#### 4.4.2. Feeding ^13^C Fatty Acid

The three feeding methods described above were also used in the field with minor modification. Fruit were selected for normal development and freedom from visual defects. Fruit hanging vertically from their spur were selected for ease of application. Cylindrical tubes were used for finite dose feeding or tubes with a tapered end and a hole in the tip for infinite dose feeding. Tubes were mounted as described above. 300 µL of donor solution containing ^13^C fatty acids were pipetted into the tubes. In infinite dose feeding, the solution was applied through the hole in the tip of the tapered tube. The hole was then sealed using silicone rubber. This setup prevented evaporation of the donor solution in the infinite dose system. In contrast, in finite dose feeding a cylindrical tube with an open top was used for feeding. Here, the donor solution evaporated within about 3 h and a dried deposit formed on the fruit surface. Unless specified otherwise, all tubes were removed after 7 d of feeding. The original footprint of the tube was marked using a permanent marker. This portion of the fruit surface was then rinsed with deionized water. Fruit were sampled 14 d after termination of the 7 d feeding period. The total time for incorporation thus was 21 d for all three feeding methods.

For feeding by perfusion, 50 µL of ^13^C fatty acid solution was directly injected into the flesh very close to the dermal tissue of the fruit using a micro-syringe. To control the depth of perfusion, the hypodermic needle was cut to 2.5 mm length. The portion of the fruit surface in contact with the injected solution was easily visible from the outside. This portion of the surface was immediately marked using a permanent marker. Subsequently, the hole in the fruit surface resulting from the perfusion was sealed with silicone rubber. Treated fruits were sampled 21 d after perfusion.

#### 4.4.3. Sample Processing and ^13^C Analysis

At the end of the feeding period, samples were fractionated in analogy to the procedure used for the ^14^C labelled samples, with minor modifications. The footprint of the fruit surface exposed to the fatty acid solution was rinsed with 1% glucopon surfactant solution and then blotted. The ES were excised from the treated surface using a biopsy punch (12 mm diameter, Acuderm, Terrace, FL, USA) and incubated in the enzyme solution as described earlier. The enzyme solution was regularly refreshed until the CMs separated from adhering tissue. CMs were cleaned using fine soft paintbrushes and rinsed with deionized water. Thereafter, CMs were dried overnight at 40 °C, weighed on a microbalance (CPA2P; Sartorius, Göttingen, Germany). The mass per unit area was calculated.

The quantity of C and the composition of regular carbon (^12^C) and stable isotopic carbon (^13^C) were measured on an elemental analyzer (Isotope Cube; Elementar, Hanau, Germany) coupled with an isotopic ratio mass spectrometer (Isoprime precisION; Isoprime-Elementar, Manchester, UK). Small pieces of dried CM (approx. 0.25 mg) were weighed into aluminum pans (6 × 6 × 12 mm^3^; LabNeed GmbH, Nidderau, Germany) and the pans crimped. The samples were burnt in the oxidation reactor of the elemental analyser at 1080 °C under a pulse of oxygen. Combustion to CO_2_ was catalyzed by the CeO_2_ filling of the oxidation reactor. The C content was quantified using a heat conductivity detector. The detector was calibrated for each measurement using a commercial sediment standard.

Online calibration of C isotopes was done by perfusion of one pulse of reference gas. Isotopic composition of C isotopes was calculated in the delta notation referenced against Vienna Pee Dee Belemnite (VPDB) for C. Further calculations of isotope mass balancing (see equations below) were done based on the at% notation. Referencing was done using international isotope standards from the International Atomic Energy Agency (IAEA, Vienna, Austria).

Sucrose (IAEA-CH-6), cellulose (IAEA-CH-3) and caffeine (IAEA-600) were used as standards for C isotopic composition. An in-house standard made of spruce litter was used as an internal standard for quality control of C composition and the referenced isotopic composition.

The relative contribution of tracer derived carbon ($R_{Tracer}$) (new carbon) to total carbon pool (old plus new carbon) was calculated using a two-pool dilution model following Gearing et al. [49] and the following equation:

$$R_{Tracer}=\;\frac{at\%\;L-at\%\;C}{at\%\;T-at\%\;C}\times 100.$$

In this equation at% represents the at% value of tracer (*at*%*T*) and labelled (*at*%*L*) or unlabelled control (*at*%*C*) CM material. Then, total mass of tracer in the whole CM sample ($M_{Tracer}$) was calculated using the following equation:

$$M_{Tracer}=\;\frac{R_{Tracer}\times \;M_{Sample}\times \;\%C}{m_{sample}}.$$

Here $M_{Sample}$ represents the total mass of sample used for the labeling procedure, *%C* the carbon content of the respective sample, and $m_{sample}$ represents the molar C-weight in the sample. All % values used in the above equations were divided by 100 prior to calculation.

Using this setup, the amounts of uptake and incorporation of ^14^C and ^13^C oleic acid into the cutin and wax fraction were quantified in developing ‘Idared’ apple fruit at 62 to 65 DAFB. Donor solutions were prepared at 200 mg L^−1^ (^14^C oleic acid) or 300 mg L^−1^ (^13^C oleic acid). The precursors were fed to the ES using the perfusion, the infinite or the finite dosing procedure described above. The ^13^C oleic acid was fed to developing apple fruit that remained attached to the tree in the field. Due to safety regulations, the ^14^C oleic acid was fed to detached fruit in the lab. The treated fruit were selected from the same trees as those used for the ^14^C treatment in the lab.

## Figures and Tables

**Table 1:** Incorporation of ^13^C or ^14^C (% of total applied) in the cuticular membrane (CM), cutin and wax of young (62 or 65 days after full bloom; DAFB) ‘Idared’ apple fruit. In vivo: Three alternative methods were used to apply ^13^C labelled oleic acid to the surface of growing fruit in the field. Incorporation was quantified by mass spectrometry. *In vitro*: ^14^C labelled oleic acid was fed to excised exocarp segments (ES) and incorporation quantified by radioactivity counting. For details see Materials and Methods. The values are means ± SEs of 8–15 replications.

			Incorporation (% of Total Applied)		
Precursor	Stage (DAFB)	Method	CM	Cutin	Wax
^13^C	65	Perfusion	4.0 ± 0.6	1.9 ± 0.3	2.1 ± 0.5
		Infinite	17.0 ± 2.0	10.1 ± 1.7	6.9 ± 1.1
		Finite	15.4 ± 2.6	10.6 ± 1.7	4.9 ± 2.0
^14^C	62	Perfusion	4.5 ± 0.3	0.5 ± 0.0	4.1 ± 0.3
		Infinite	19.5 ± 1.1	14.5 ± 1.0	5.1 ± 0.2
		Finite	16.3 ± 0.7	7.5 ± 0.5	8.8 ± 0.3

## Data Availability

Original data is available upon request from the corresponding author.

## Supplementary Materials

The following are available online at https://www.mdpi.com/2223-7747/10/3/497/s1, Figure S1: Sketch illustrating three different feeding methods for the ^14^C labelled fatty acids used in the in vitro laboratory assay. In finite and infinite dose feeding, a polyethylene (PE) tube is mounted on the skin of the epidermal segment (ES). The donor solution containing the fatty acid is pipetted into the PE tube. In finite dose feeding the donor solution dries leaving behind a dried down deposit containing the fatty acid on the skin. In infinite dose feeding, the donor solution is prevented from drying. The fatty acid stays in solution throughout the feeding period. In contrast, in perfusion feeding, the donor solution is applied onto the cut surface of the ES.
