# Supplementary material for: Cutin Synthesis in Developing, Field-Grown Apple Fruit Examined by External Feeding of Labelled Precursors
Source: Plants (Basel). 2021 Mar 5;10(3):497. doi: 10.3390/plants10030497 (PMC8000455; doi:10.3390/plants10030497)
Supplement: Supplementary file 1 [file plants-10-00497-s001.pdf]

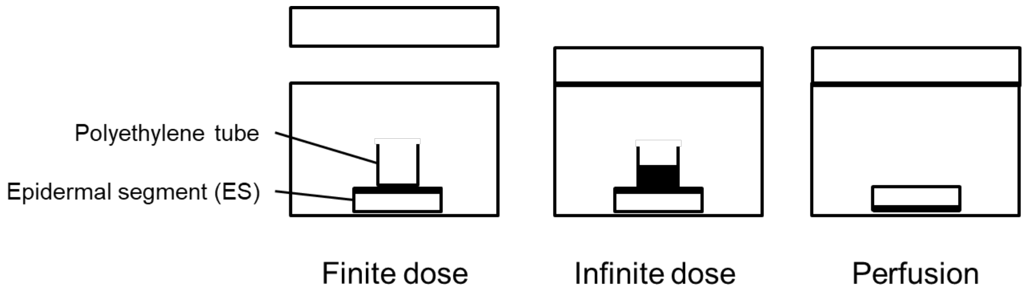

Figure S1. Sketch illustrating three different feeding methods for the ^{14}C labelled fatty acids used in the *in vitro* laboratory assay. In finite and infinite dose feeding, a polyethylene (PE) tube is mounted on the skin of the epidermal segment (ES). The donor solution containing the fatty acid is pipetted into the PE tube. In finite dose feeding the donor solution dries leaving behind a dried down deposit containing the fatty acid on the skin. In infinite dose feeding, the donor solution is prevented from drying. The fatty acid stays in solution throughout the feeding period. In contrast, in perfusion feeding, the donor solution is applied onto the cut surface of the ES.
